# Exploring the Mechanical Properties and Performance of Type-I Collagen at Various Length Scales: A Progress Report

**DOI:** 10.3390/ma15082753

**Published:** 2022-04-08

**Authors:** Shirsha Bose, Simin Li, Elisa Mele, Vadim V. Silberschmidt

**Affiliations:** 1 Wolfson School of Mechanical, Electrical and Manufacturing Engineering, Loughborough University, Loughborough LE11 3TU, Leicestershire, UK; s.bose@lboro.ac.uk (S.B.); s.li@lboro.ac.uk (S.L.); 2 Department of Materials, Loughborough University, Loughborough LE11 3TU, Leicestershire, UK; 3 Laboratory of Mechanics of Biocompatible Materials and Devices, Perm National Research Polytechnic University, 614990 Perm, Russia

**Keywords:** collagen, hierarchical organisation, hydration, long-term mechanical performance

## Abstract

Collagen is the basic protein of animal tissues and has a complex hierarchical structure. It plays a crucial role in maintaining the mechanical and structural stability of biological tissues. Over the years, it has become a material of interest in the biomedical industries thanks to its excellent biocompatibility and biodegradability and low antigenicity. Despite its significance, the mechanical properties and performance of pure collagen have been never reviewed. In this work, the emphasis is on the mechanics of collagen at different hierarchical levels and its long-term mechanical performance. In addition, the effect of hydration, important for various applications, was considered throughout the study because of its dramatic influence on the mechanics of collagen. Furthermore, the discrepancies in reports of the mechanical properties of collagenous tissues (basically composed of 20–30% collagen fibres) and those of pure collagen are discussed.

## 1. Introduction

Collagen is a structural protein, which is of upmost importance to vertebrates, as it contributes to one-third of their body mass [1,2]. It is responsible for mechanical stability and structural integrity of living organisms, and is prevalent in both mineralised (bone, teeth, fish scales) and nonmineralised (skin, tendon, ligament, cornea) tissues. At present, 29 types of collagens have been identified [3], with Type I collagen being predominant [4], constituting the extracellular matrix (ECM) of human tissues such as skin, tendons, etc. Considering its ubiquity, mechanical importance, and applications, over 2000 research articles have been published on the mechanical behaviour/properties of various collagen-rich native tissues and engineered collagenous materials since 2011. The literature search for this review was conducted on Scopus using the keywords “collagen”, “mechanical”, and “type I”.

To date, research on collagen-based materials has mainly focused on applications, fabrication, manufacturing, and morphological analysis [5,6,7,8,9]. Very few attempts have been made to review the latest advances on the mechanics of collagenous materials [9,10]. Though several studies have been performed at molecular and fibrillar level, the mechanics of collagen at higher levels of hierarchy have been explored only to a limited extent. This review introduces the hierarchical organisation of collagen followed by discussion on engineered collagen structures used for biomedical applications. The main objective of this review is to highlight the state of the art of the mechanics of collagen at different length scales over the past decade. Another important factor—the influence of hydration—was incorporated because it significantly impacts collagen mechanical behaviour. In addition, this paper analyses the misconception in assessment of the mechanical response of collagenous tissues and the long-term mechanical performance of collagenous engineered materials. The focus is on type I collagen because of its high relevance to the biomedical field. Here, pure collagen refers to collagen without any additives, in the case of processed collagen, or substances such as proteoglycans, in the case of native tissues.

### 1.1. Hierarchical Organisation of Collagen

**Figure 1:**
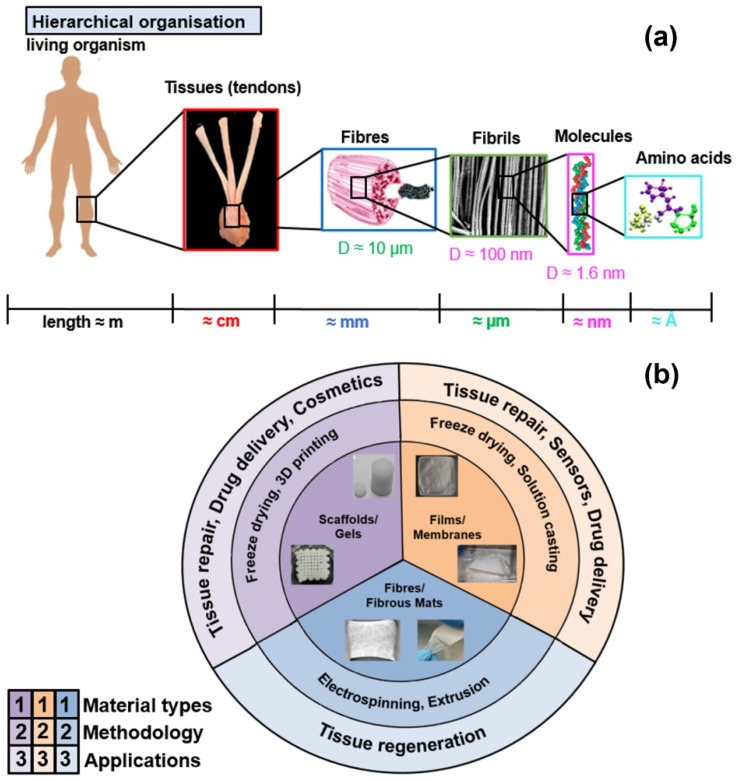
(**a**) Hierarchical organisation of collagen at different length scales [9,31,32,33]. (**b**) Various collagen structures [18,25,26,30,34,35] with the corresponding processing/fabrication techniques and biomedical applications.

The hierarchical organisation of collagen is a widely investigated feature because it impacts on the properties of both collagenous tissues and engineered collagen structures. At the lowest level of hierarchy (Figure 1a), collagen consists of three left-handed polypeptide chains, which coil up to form a right-handed triple-helix structure—tropocollagen (collagen molecules). Each polypeptide chain, in turn, is formed by an amino acid motif: Gly-X-Y, where Gly represents the glycine (core protein) and X and Y are the amino acids. The molecules have a length and diameter of 300 nm and 1.5 nm, respectively [11]. These molecules are arranged in a special manner to form a periodic structure, a D-band (D = 67 nm [12]), with alternating gap (0.46 D) and overlap (0.54 D) regions [13]. The next level in the hierarchy is represented by collagen fibrils (diameter of 100–500 nm; length of a few millimetres), consisting of five collagen molecules placed in a staggered manner. The fibrils combine to form the collagen fibres, which are constituent parts of various tissues and organs (e.g., tendons, ligaments, skin) and determine their mechanical strength [14,15]. Tissues are at the highest level in the hierarchy (Figure 1a) and form living organisms (human body).

### 1.2. Engineered Collagen

Over the past decade, numerous technologies have been successful in manufacturing various engineered collagenous structures for specific biomedical applications. The most widely used natural sources of collagen include bovine, porcine, rodent, and human tissues [5,16]. Frog and sheep skin, avian (for example, chicken and duck feet) and alligator bones, kangaroo tails, and equine tendons are also used. Recently, marine collagens extracted from fish scales, jellyfish, etc. have attracted increasing interest as they do not transmit infectious diseases such as zoonosis [17,18]. Though collagens extracted from natural sources are clinically approved, there might be some batch-to-batch variability, and they can induce inflammation during applications [19,20,21,22]. Synthetic collagen, commercially known as KOD, is an alternative to natural collagen and consists of 36 amino acids that self-assemble to form triple-helix nanofibers and hydrogels with minimal inflammatory production [22,23]. Recombinant technology produces stable collagen with specific DNA expression, intact triple-helix configuration, low immunogenicity, biocompatibility, and biodegradability [24]. However, the high cost associated with this approach is a major limitation for the widespread availability of synthetic collagens [16]. Thus, the extraction of collagen from natural sources is still the most popular strategy.

Numerous technologies have been successfully employed to develop various engineered collagenous structures such as gels, sponges, membranes/films, fibrous mats, intricate 3D scaffolds, hollow tubes, etc. [5]. One of the most extensively used methods is freeze-drying [18] to produce membranes, 3D gels, and scaffolds by freezing a collagen solution and then sublimating the ice. Fibrous collagen sheets and mats can be produced using electrospinning, which enables control over fibre diameter [25]. Recently, additive manufacturing has been widely acknowledged to achieve precise collagen deposition, incorporate bioactive compounds, control mechanical properties, and achieve cost effectiveness [26]. Some other technologies for collagen processing are extrusion of fibres [27] and filaments [28] and electro-compaction [29]. Collagen manufactured with these techniques finds a wide range of biomedical applications, including tissue regeneration [5] and, recently, substrate for flexible electronics [30]. The most commonly available engineered collagen structures, along with their most relevant applications, are shown in Figure 1b. Structural arrangements at different length scales affect the multiscale mechanical performance of collagen constructs.

## 2. Mechanical Behaviour at Different Length Scales

**Figure 2:**
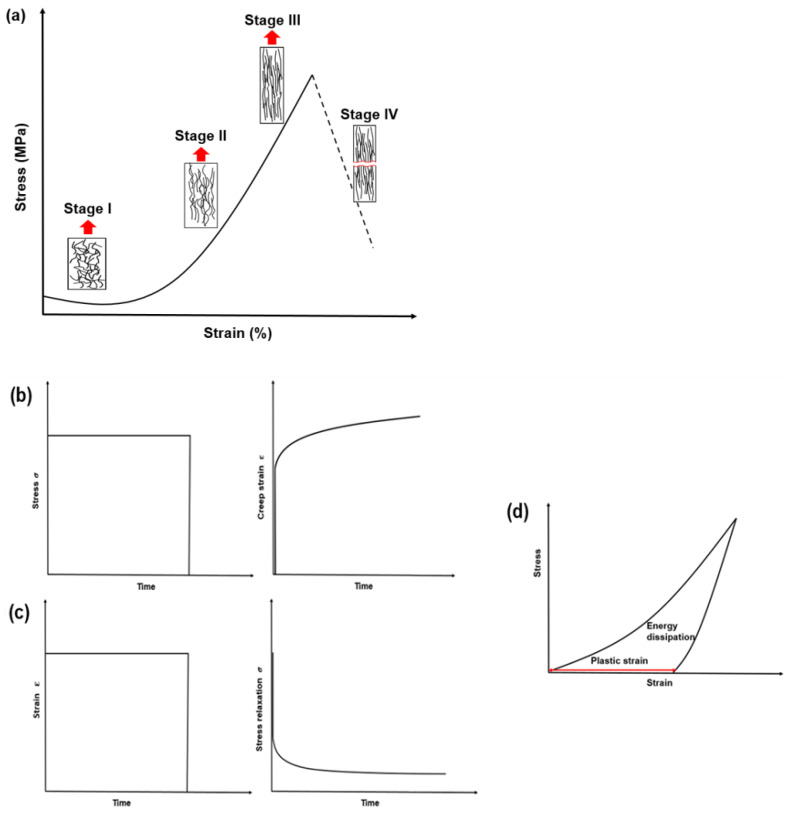
(**a**) Schematic representation of the arrangement of collagen fibres with increasing strain during tensile loading resulting in stiffening. Viscoelastic effects: (**b**) creep; (**c**) stress relaxation; and (**d**) energy dissipation in cyclic loading (only one cycle shown).

The mechanical responses of collagen-rich materials have been discussed and compared widely in various previous works [14,36,37]. Soft collagenous tissues, such as skin [38] and arteries [39], are known to exhibit a strain-stiffening behaviour—also referred to as J stress–strain curve (Figure 2a). The stiffening mechanism becomes prominent at higher strain levels because of recruitment and elongation of the embedded collagen fibres. The main stages of this tensile response include: (i) random orientation of collagen fibres; (ii) recruitment and straightening of collagen fibres along the tension direction; (iii) sliding, stretching, and delaminating of fibres and their alignment along the tension direction (strain stiffening); and (iv) fracture of the fibres [40].

Collagen-rich materials have shown time-dependent viscoelastic behaviour in both soft and mineralised collagen materials. This was quantified at various levels of hierarchy using cyclic loading, creep, and stress-relaxation behaviours (Figure 2b–d). During creep, the stress is kept constant while the increase in the strain is recorded (Figure 2b); for stress-relaxation, the strain is kept constant, and the decreasing stress is noted over time (Figure 2c). In cyclic loading, the energy dissipation is estimated from the area under the loading–unloading curve. It was observed that collagenous tissues accumulated plastic strain (ratchetting behaviour) with increasing cycles [36,41,42]. The increase in creep strain and the exponential decay of stress during relaxation tests indicated the viscous nature of collagen [14,41,42,43,44,45]. Although these properties have been well reported in the literature, still, only limited discussion is available on the mechanics of collagen at various length scales considering a hierarchical organisation ranging from nanometres to meters. The next section focuses on the advances in this area over the last decade.

Several experimental techniques and instruments have been used to measure the mechanical properties of collagen at different length scales. Conventional nanoindentation and atomic force microscopy (AFM) use sharp probes to estimate the elastic modulus of the materials, which depends strongly on the indentation depth [46]. Generally, conventional nanoindentation has a limitation—it does not offer loads in a lower range, which are necessary to test soft biomaterials. AFM can provide data on both surface roughness and forces, and it is suitable to test soft materials. Hence, imaging and mechanical behaviour of the material can be obtained in a single test [47]. However, the use of AFM has several limitations, including the effects of indenter geometry [48], the thickness of the specimen [49], and overestimation of the elastic modulus due to the indentation depth [50,51,52], linked to the indenter’s radius [53]. On the other hand, microelectromechanical systems (MEMS) were developed to measure the mechanical properties of single collagen fibrils in tension, while AFM generally measures their radial rather than their axial properties [43]. Still, some studies have also quantified tensile properties with AFM at nanoscale [15,54]. Initially, MEMS devices used electrostatic actuation to apply a force, and the corresponding deformation was measured with a vernier scale. However, currently, they employ piezoelectric measurements of displacements and are capable of recording nanoscale displacements when equipped with digital image correlation (DIC) software [55]. Although significant advancement was achieved in the past years, errors in the mechanical response related to the device setups might occur [56]. Traditional tensile tests have been used to characterise the mechanical properties of collagen at macroscale and estimate its bulk modulus. Although they provide a more robust, repeatable, and scalable method to characterise mechanical properties compared with those discussed above, defects (such as pores) in the material can affect the analysis, leading to premature failure of the specimen.

### 2.1. Molecular Level

The elastic properties of single collagen molecules were widely explored over the past decades by utilising both experimental and simulation techniques. Although it was recognised that collagen-rich tissues exhibited viscoelastic responses [37,57], the underlying justification (at molecular levels) was not established until the last decade. The mechanisms involved in the nonlinear viscoelastic response of single collagen were reported for the first time by Gautieri and co-workers [58] using an atomistic modelling (AM) approach. They demonstrated that the elastic components of collagen molecules originated from stretching, while water molecules contributed to the viscous response. The viscoelastic behaviour of collagen constructs was attributed to the interplay of various molecular mechanisms, such as molecular stretching (dry fibrils) and sliding (wet fibrils) [59]. The viscous behaviour was also evident during wave propagation, which resulted in higher (almost twofold) energy dissipation for hydrated materials than for dry ones, the increase being attributed to the presence of water molecules [60]. The magnitudes of global and local mechanical properties are functions of the applied hydrostatic pressure—revealing the orthotropic nature of collagen molecules [61,62]. Initially, the AM approach was used to model shorter segments of such molecules [58,63], with later attempts made to model larger segment lengths [64] with an interconnecting H-bond [64,65]. An increase in the segments (to 290 nm) resulted in a linear growth of creep strain [64]. Advanced computational models predicted the mechanical response of the molecules in the in vivo environment [65]. Advancement in computational studies has unveiled various aspects and underlying mechanisms of collagen at the molecular level. Still, despite much effort, the effects of cross-linking and damage-related interactions affecting the mechanical behaviour of tissues (macroscale level) have not been investigated at the molecular level. The corresponding variances might be strongly related to the molecular level, just as molecular mechanisms are responsible for the viscoelasticity of the fibrils and tissues.

### 2.2. Microfibril/Fibrillar Level

The mechanical response of a collagen fibril can be estimated using both experimental and modelling (AM) approaches, though it is challenging to simulate millions of atoms. The experimental quantification of the mechanical properties of a single fibril has been extensively studied with tensile testing using MEMS [44,66] and AFM [15,67,68]. Earlier research on single fibrils was limited within the elastic regime and provided the corresponding elastic modulus [67] in various media [67,69,70]. With the advancement of MEMS devices, more robust testing in tension was performed to analyse the fracture stress of a single fibril [71]. A time-dependent study on a single fibril showed intrinsic viscoelastic behaviour with an initial lower relaxation time (7 ± 2 s) followed by a stage with longer time (102 ± 5 s) [43]. Further investigations were performed to characterise the viscoelastic properties of the collagen fibril by assessing the hysteresis, cycling, and strain-rate-dependent behaviour. These clearly demonstrated the contribution of the viscous component due to molecular and microfibrillar sliding [15,44]. These mechanisms are also responsible for the failure of pure (nonmineralised and non-cross-linked) collagen fibrils during high strain levels [44,72,73]. Other studies showed that collagen fibrils (tendon) were rather mechanically continuous, suggesting that their failure originated from fibrillar breakage and molecular stretching [72,73,74,75] rather than slippage. Cycling loading can induce damage in the form of plastic deformation or discrete plasticity and fatigue damage (kink bands in AFM images) in certain fibrils depending on their location in the body [75,76].

AFM-based indentation has produced some promising results in the last decade with the implementation of specific testing protocols [52]. Values obtained for the modulus of both rat tail tendon (3.2 ± 1.1 GPa) and human bronchial biopsies (6.6 ± 0.7 GPa) were in agreement with previously published results. Later, Gachon and Mesquida studied the effect of the increased depth of AFM-based nanoindentation (by 25%) in collagen fibrils with a biphasic mechanical response—strain hardening (up to 15%) followed by a softening behaviour (up to 25%) [68]. The same group showed the charging of a fibrillar surface induced by high mechanical strains. Hydration plays a significant role in the structure–mechanics relationship of collagen. A study by Masic et al. [77] revealed a reduction in the gap/overlap region of a D-band upon dehydration accompanied by generation of high levels of stresses (up to 100 MPa). The mechanics of fibrils highly depend on the extent of hydration [78], with a loss of bound water (dehydration) leading to shrinkage of fibrils and a corresponding effect on their mechanical properties [79]. Hydrated fibrils demonstrated an increase in stiffness upon stretching that might be associated with a higher D-band length [80].

Although experimental studies have been performed on single collagen fibrils, molecular simulations are still essential to comprehend the interactions and deformations occurring at the molecular level that contribute to multiscale collagen mechanics. These simulations are challenging, as they involve several millions of molecules (each molecule around 300 nm in length), resulting in a high computational cost [9]. Over the last decade, the simulations have been improved, incorporating microfibril/fibrillar segments using a bottom-up atomistic approach [58,63,81] for various types of cross-links [72]. Molecular dynamics have also been employed to analyse the variation in fibrillar mechanics during degradation [66] and the effect of hydration on both nonmineralised and mineralised fibrils [67]. With further advancements in computational technology, it is expected that, in the upcoming decade, AM could be used as a tool, at the molecular and fibrillar levels, to investigate the mechanical performance and structural integrity of tissues affected by diseases (such as diabetes [66], osteoarthritis, and osteoporosis).

The variance in the obtained magnitudes of the modulus of the fibril (see Table 1) highly depended on several factors, such as the effect of hydration and simulated conditions [59,71,82,83], the degree of cross-linking [54], the source of the fibril [84], and the extent of mineralisation [82,85]. Water molecules act as a spacer in the collagen molecules, leading to formation of H-bonds between the amide and carbonyl groups and thus contributing to the mechanical behaviour of collagen fibrils [39]. Such changes in the modulus are dependent not only on hydration but on variation in pH levels [70]. Apart from the physiochemical factors, mechanical cues are also responsible for the specific features of fibrillar mechanics. Increased stiffness in the fibrils was observed after repeated cycling [44,86]. Studies by An et al. [87] and Karunaratne et al. [88] revealed the strain-rate sensitivity of the fibrils, associated with their debonding, ultimately leading to the failure at the macroscopic levels.

Among the hierarchical levels of collagens, the mechanics of fibrils have been extensively studied. Nevertheless, the studies associated with the failure and fracture mechanics of fibrils need additional attention. These investigations would be beneficial to prevent tissue injuries and design artificial tissues. Both advanced experimentation and molecular simulation techniques should be used, particularly to investigate the mechanics of diseased fibrils.

### 2.3. Microscale Level (Fibres/Bundle of Fibrils)

The microscale level of collagens is dominated by the bundles of fibrils known as collagen fibres. Rather limited quantification of the mechanical properties of isolated (or “pure”) collagen fibres has been performed so far. Generally, studies have focused on macroscale tissues, such as bone and skin, that contain a certain fraction of collagen fibres [15,89,90,91]. Preliminary research was focused on the tensile properties of native and cross-linked collagen fibres [92,93], while later investigations included analysis of the mechanical behaviour of both native and extruded (synthetic) fibres. It was observed that the mechanical properties of cross-linked extruded fibres were improved, with typical J stress–strain curves and time-dependent behaviour [94] commonly observed in biological tissues due to the recruitment of collagen fibres [95].

The nanoindentation technique can be used to quantify transverse mechanical properties of collagen fibres. For collagenous tissues, it was observed that the nanoindentation modulus was much higher (63 ± 4 MPa) than that measured using other testing methods (such as tensile modulus) [96]. Panwar et al. [97] demonstrated variation in the modulus of collagen fibres from 3.20 ± 0.68 GPa (control) to 1.90 ± 0.65 GPa (cathepsin treated) resulting from the destruction of components such as proteoglycan-GAG (glycosaminoglycan). This approach was also applied to estimate nano- and micromechanical properties of collagen fibres in vaginal tissues, and major changes in elasticity and microstructure were revealed in pre- and postmenopausal women [89]. The relatively lower level of indentation modulus in the whole tissue (5–6 kPa) than in collagen fibres (80–200 kPa) resulted from the complex orientation of collagen I, smooth muscles, and extracellular (elastin and nonfibrous proteoglycans) elements embedded in the matrix. Some attempts were made to improve the manufacturing techniques and the quality of artificially engineered collagen fibres applied in tissue regeneration [98,99].

Interestingly, the mechanics of isolated collagen fibres have not been quantified in depth to date. Although several attempts have been made to assess the behaviour of collagen fibres embedded in skin, arteries, tendons, etc., the obtained results were influenced by the neighbouring matrix components, such as proteoglycans and GAGs. The major problem is that time- and frequency-dependent behaviours, which are strongly associated with the tissue mechanics and failure mechanisms have not been explored at this level of hierarchy.

### 2.4. Macroscale Level

The macroscale level of collagen is represented by tissues composed of collagen fibres and fibrils, such as skin, tendons, cornea, bones, etc. Generally, this level of hierarchy can be subdivided into mineralised (bone, dentine) and nonmineralised (skin, arteries) collagen-rich tissues. However, native tissues contain other components, such as elastin, proteoglycans, etc., along with collagen fibres. Svensson et al. [15] used the rule of mixture to estimate the occurrence of collagen (22%) in native tendon, thus reducing the overestimation of the modulus. Several other studies [89,90,91,100,101] also revealed the importance of other components (such as GAGs, elastin, proteoglycans, and enzymatic and nonenzymatic cross-links) contributing to the mechanical response at higher levels of hierarchy.

Only rather limited research has been performed to quantify the mechanical features of pure collagen at macroscale. It should be taken into consideration that collagenous tissues are composed of different ground substances; hence, pure collagen engineered constructs might be used to quantity mechanical properties. Recently, studies by Bose et al. [35,45] quantified the tensile, elastic, and time-dependent mechanical properties of collagen films in both dry and wet environments. Collagen at macroscale exhibited a rate-dependent hardening behaviour [35], with significant variation in the viscous component when the testing environment was changed from dry to wet [45]. Considering the application of engineered collagen for tissue regeneration, it is beneficial to characterise the mechanical response of collagen employing nano- and micromechanical forces. A recent study by McManamon et al. [102] used indentation to investigate the micromechanical properties of collagen-based films and showed the viscoelastic response for dry (with a modulus of 1 GPa) and wet (0.006 GPa) environments. Furthermore, recent experimental analyses of the fracture behaviour of pure collagen at macroscale using tensile loading showed that hydration strongly affected both the toughening process and resistance to crack propagation [103]. Collagen films have been used to quantify the mechanical response of collagen at macroscale because they can be manufactured in a reproducible way that enables testing in different conditions. Other types of materials, such as electrospun mats or 3D printed gels, could present some challenges, particularly if tested in an aqueous environment where collagen could be relatively weak [35]. Another study [104] demonstrated the importance of collagen fibres in maintaining the structural integrity of a temporomandibular joint under compression indentation tests.

The magnitudes of the modulus of collagen at various levels of hierarchy (both dry and wet environments) are reported in Table 1. In brief, striking deterioration was observed for the modulus of collagen tested in a wet environment when compared to that tested in a dry environment. This is due to the plasticising effect of water molecules, stemming from the reduction in the intramolecular H-bonds, defining the backbone rigidity [38]. It should also be taken into consideration that, as the length scale increased, the average modulus of the collagen decreased (as shown in Table 1). Surprisingly, only limited investigations have been performed to understand the mechanics of pure collagen at higher length scales, such as macroscale [35,45,102,103]. At the fibrillar level, the time-dependent properties still remain relatively unexplored. They would be of considerable interest for interpreting the interactions between fibrillar bundles and their contribution to the load-bearing process of the whole structure. Generally, these length scales are targeted for biomedical applications; hence, a proper understanding of the mechanics is beneficial for the design of collagen-based products for next-generation applications [105,106]. Various experimental methods used to quantify the mechanical behaviour of collagen at different length scales are listed in Table 1.

## 3. Long-Term Mechanical Performance

In the last decade, several techniques [109] have successfully demonstrated fabrication of collagen-based constructs that can guide the behaviour of cells for the regeneration of different tissues [110,111]. When implanted into the body, such constructs are exposed to in vivo conditions, which can result in considerable changes in their mechanical behaviour even within a few weeks of implantation [112,113,114,115]. During this degradation process, the constructs might not be capable of exhibiting the mechanical strength necessary for tissue repair. Therefore, it is important to monitor the mechanical properties of the constructs over time, referred to as the long-term mechanical performance in this section. Furthermore, the constructs should be exposed to physiological conditions, typically represented by immersion in a phosphate-buffered solution (PBS) at 37 °C.

The rapid degradation of engineered collagen structures is usually evident from the high weight loss after exposure to in vivo conditions for a few days, as presented in Table 2. As discussed, this degradation severely affects the mechanical performance of the constructs. To date, most studies have analysed the deterioration of these constructs considering degradation kinetics and weight loss with increasing time (in weeks). It is also expected that the morphology of such structures can change with time. However, only limited research [116,117] is available on the mechanical and morphological characterisation of the structures along with their weight loss. Examples include collagen tubes tested after one day of exposure to the medium [118] and cross-linked collagen scaffolds over 14-day exposure to various bodily fluids [117]. Cross-linking was used to enhance the mechanical stability of these scaffolds [119] in conditions reproducing the in vivo environment. A study by Bose et al. [116] showed the rapid degradation of the tensile mechanical properties of collagen films (by about 26% within 10 days of exposure); it was impossible to acquire any data on the day 14 of water exposure because of the advanced degradation of specimens causing their loss of stability. Though this study demonstrated the change in the morphology with time, there was still no investigation of the effect of collagen’s dissolution in enzymatic environment and its corresponding long-term mechanical stability. Overall, it can be concluded that long-term mechanical performance of collagen structures needs further analysis in an appropriate testing environment mimicking that in vivo. This is a major aspect to consider, as the high degradation rate of collagen can limit its uses.

Apart from the collagenous constructs, the structural and mechanical properties of the native tissues are also affected by increasing age and diseases [131,132]. The role of the collagen phase is crucial for mineralised tissues (bone), as it provides toughness to a composite system made of a brittle phase (mainly hydroxyapatite) and a soft, collagenous matrix [133]. Investigations of the mechanics of the collagen phase revealed that its mechanical strength and modulus [134] decreased because of deterioration of collagen fibrils [135]. Remodelling of the bone leads to variation in the collagen fibre orientation along with an increase in denatured collagen (with age), affecting the bone’s mechanical properties [136]. Furthermore, the formation of both enzymatic and nonenzymatic collagen cross-links caused by age and disease-related factors severely affects the bone, making it brittle [137]. Another aspect to consider is the interface between hard and soft tissues, for example, articular cartilage and tendon-to-bone and -muscle interfaces. Here, a complex organisational structure and a mismatch in the mechanical response pose challenges in creating artificial constructs such as functionally graded materials that can provide consistent gradient formation, mechanical support, and biophysical properties [138].

The load bearing capacity of a nonmineralised tissue (such as skin, artery) is highly dependent on its collagen component. Interestingly, the role of the collagen phase in different nonmineralised tissues varies significantly, for example, with age. In old tissues, the structural organisation of collagen lacks fibrillar/fibre orientation [131,139]. During the ageing process, stiffness increases because of the formation of enzymatic collagen cross-links (originating from amino acids) or nonenzymatic advanced glycation end products [139,140,141,142]. The nonenzymatic cross-links are mainly due to the accumulation of glucose (resulting from diabetes) over time, which leads to the formation of collagen cross-linking in both nonmineralised and mineralised tissues. This brief discussion on the long-term mechanical performance of native collagen-rich tissues reveals the importance of collagen for maintaining the mechanical and structural stability of tissues. The change in tissue mechanics over time has been extensively explored over the past decade.

## 4. Conclusions

The report highlights the major achievements in analysing the mechanics of pure collagen at different hierarchical levels in the last decade. Molecular sliding originating at the lower length scales (molecules and fibrils) was responsible for the viscoelastic response of collagen-rich tissues. Although the mechanical characterisation of collagen molecules, microfibrils, and fibrils has been extensively performed with both advanced experimental and simulation techniques, only limited research has focused on the mechanics of collagen at higher length scales (such as pure collagen fibres and bundles of fibres), as recognised in various works. The study of reversibility (loading–unloading) should be performed for collagen fibres, as various tissues are exposed to cyclic loading. The energy dissipated by the whole tissue is transferred to fibres and then relayed to the fibrils and molecules. This complex transfer of viscoelasticity from the higher levels to the lower levels needs attention, since it could aid in the processing of collagenous constructs. Additionally, detailed studies of the mechanics of collagen constructs considering the mechanisms of stress transfer from the higher to the lower levels of hierarchy would enhance our understanding of the in vivo behaviour of these materials. Moreover, without proper investigation of the mechanical behaviour at the macroscale level, it would be rather challenging to determine and elucidate the mechanisms underpinning the contribution of the collagen fraction to the time-dependent viscoelastic response of native tissues.

In addition, in this review, the long-term mechanical performance of engineered collagen constructs is discussed. Numerous studies have demonstrated the variation in stiffness and ductility of aged and diseased collagen-rich tissues and fibrils. However, very limited attempts have been made to assess the mechanical performance and underlying mechanisms of rapid degradation of engineered pure collagen. Proper investigations would help with the development of collagen scaffolds with controlled properties for specific biomedical applications.

Future research could aim to improve the understanding of the mechanics of pure collagen at all hierarchical levels when exposed to the in vivo environment. This would assist the development of advanced treatments for diseased tissues and next-generation scaffolds for tissue engineering.

## Figures and Tables

**Table 1:** Extent of deformation and modulus for dry and wet collagen at all hierarchy levels.

Hierarchical State	Analysis Method	Extent of Deformation		Modulus (GPa)		Ref
		Dry	Wet	Dry	Wet	
**Molecular level**						
Long, short molecule	MD and SMD	—	15–25%	—	4.6, 6.2	[63]
Single molecule	AM	—	20%	10–19	6–16	[58]
Six-molecule segments	AM	—	10.5–12.5%	—	3.2–4.9	[65]
Collagen molecular segments	AM	—	<40%	—	4.75 ± 0.045	[64]
Collagen peptide	AM	10%	10%	15.21	5.85	[60]
**Fibrillar level**						
Single fibril	MEMS	—	30%	—	0.12 ± 0.0046	[55]
Isolated fibril	AFM (nanotensile test)	0.86 ± 0.08%	—	2.8 ± 0.3 (LM)	1.0 (LM)	[15]
Rat tail fibril	AFM (nanoindentation)	<10%	—	3.2 ± 1.1 (LM)	—	[52]
Human fibril	AFM (nanoindentation)	<10%	—	6.6 ± 0.7 (LM)	—	[52]
Isolated fibril	MEMS	—	86%	—	0.33 ± 0.11	[43]
Microfibrils	AM	High strain	High strain	2.25	1.2	[32]
	AFM (nanotensile test)	—	13 ± 2%	—	0.6 ± 0.2 (LM)	[54]
Microfibril assembly	AM	—	—	—	2.24-3.27	[81]
Fibrils (connected by covalent bonds)	AM	—	—	9	2.5	[59]
Single human fibril	AFM (nanoindentation)	—	—	2-4	—	[107]
Mineralised collagen microfibril	AM	—	<4%	—	2.38 ± 0.37	[85]
Single fibril	MEMS	—	Low strain	—	4.3 ± 1.1	[44]
**Fibre level**						
RTT fibres (non-cross-linked)	TT	16–18%	6–7%	2.1–2.7	0.47–0.57	[93]
Single RTT fibre (cross-linked)	TT	—	—	—	1.17	[94]
Extruded fibre (cross-linked)	TT	—	—	—	0.27–0.50	[94]
Collagen fibres (control)	TT	39 ± 7%	—	3.21 ± 0.68	—	[97]
Collagen fibre (18-tendon calf)	Nanoindentation	—	—	—	0.06 ± 0.004	[96]
Extruded fibre (cross-linked)	TT	—	21–25%	—	0.018–0.05	[98]
Extruded fibre (non-cross-linked, nonmineralised)	TT	11 ± 8%	31 ± 12%	2.34 ± 0.63	0.0047 ± 0.0011	[99]
**Macroscale level**						
Pure collagen film	TT	23.5 ± 2.5%	55.9 ± 2%	1.0–1.2	0.0017–0.014	[35]
Pure collagen scaffold	TT	—	—	—	0.005–0.04	[108]
Collagen—hyaluronic film	Indentation	—	—	1.0	0.006	[102]
Pig skin (60–80% collagen, dry tissue weight)	TT	—	20–45%	—	0.04–0.085	[14]
Tendon (22% collagen)	Ultrasound	—	3.3 ± 1.9%	—	2.0 ± 0.05	[15]
Bovine cortical bone (30% collagen by volume)	TT	—	0.4–1.1%	—	10–24	[36]

MD—molecular dynamics; SMD—steered molecular dynamics; AM—atomistic modelling; MEMS—microelectromechanical system; AFM—atomic force microscopy; TT—tensile testing; LM—longitudinal modulus. Parameters in the italics are for collagen-containing tissues.

**Table 2:** Long-term performance of pure engineered collagen (with increasing period of exposure in days).

Structure	Exposure Medium	Exposure Period (Days)	Long-Term Performance			Remarks	Ref
			Morphological Analysis	Degradation Kinetics/Weight Loss	Mechanical Testing		
Electrospun fibres	Deionised water, 37 °C	0.08	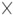	22%	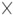	Severe enzyme degradation; neither hydrated nor immersed in solution before mechanical testing	[120]
Scaffold (non-cross linked)	Collagenase degradation	0.25	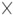	Almost completely digested	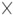	Non-cross-linked scaffolds were highly digested by collagenase	[112]
Film	Collagenase, 37 °C	0.25	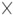	23%	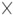	Mechanical stability not characterised	[121]
Film (non-cross-linked)	Enzymes, 37 °C	1	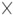	Completely degraded	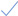	Mechanical characterisation conducted with samples exposed to PBS, 37 °C for 1 day	[113]
Tube	Deionised water, 37 °C	1	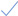	70%	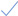	Nanofibers dissolved, showing smooth surface, after 1 day of immersion	[118]
Hydrogel	PBS, 37 °C	3	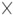	35%	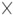	High permeation due to larger pore size	[122]
Film	PBS, 37 °C	3	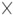	90%		Mechanical test performed after 10 days of in vitro insertion	[123]
Scaffold	PBS, 37 °C	6	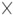	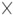	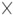	Long-term properties of pure collagen not investigated	[124]
Scaffold (uncross linked)	PBS	14	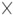	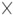	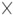	Dramatic difference in properties of non-cross-linked and cross-linked collagen scaffolds	[125]
Scaffold (cross-linked)	Human blood plasma, PBS/SBF, 37 °C	7/14	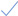	5–15%/5–25%	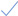	Cross-linking did not necessarily determine properties of pure collagen; no information on environment of mechanical testing	[117]
Film	Water, 20 °C	14	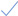	30.5% ± 5.6%	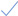	Enzymatic degradation not considered	[116]
Scaffold	PBS or DMEM, 37 °C	15	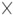	80%	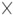	Higher degradation for in vitro implantations	[126]
Scaffold	PBS, 37 °C	18	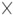	Completely dissolvable	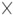	No long-term mechanical characterisation	[114]
Scaffold	PBS, 37 °C	21	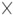	Completely dissolved	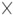	Mechanical performance tested after 1 day of immersion	[115]
Hydrogel	PBS	28	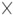	86%	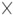	High water retention capability	[127]
Hydrogel	PBS, 37 °C	28	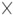	75–80%	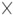	High shrinkage during cell-culture exposure	[128]
Film	Distilled water, 37 °C	28	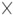	15%	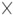	Cell adhesion showed stability on day 7 of seeding	[129]
Scaffold	PBS, 37 °C	28	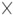	98%	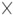	Specimen neither hydrated nor immersed in any solution before mechanical testing	[130]
Two-ply yarn	0.01M PBS, 37 °C	56	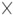	61.8% ± 4.5%	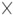	No mechanical testing for degraded sample	[28]

PBS—phosphate-buffered solution, SBF—stimulated body fluid, DMEM—Dulbecco’s modified eagle medium. The symbol 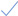 indicates that the corresponding analysis was performed, while the symbol 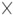 denotes that it was not.

## Data Availability

Not applicable.
